# Time Dependent Behaviour of Trabecular Bone at Multiple Load Levels

**DOI:** 10.1007/s10439-017-1800-1

**Published:** 2017-01-27

**Authors:** Shuqiao Xie, Krishnagoud Manda, Robert J. Wallace, Francesc Levrero-Florencio, A. Hamish R. W. Simpson, Pankaj Pankaj

**Affiliations:** 1 Institute for Bioengineering, School of Engineering, The University of Edinburgh, King’s Buildings, Edinburgh, EH9 3DW UK; 2 Department of Orthopaedics, The University of Edinburgh, Chancellor’s Building, Edinburgh, EH16 4SB UK

**Keywords:** Creep-recovery, Viscoelastic, Bone volume fraction, Steady state creep rate, Creep compliance

## Abstract

The deformation of bone when subjected to loads is not instantaneous but varies with time. To investigate this time-dependent behaviour sixteen bovine trabecular bone specimens were subjected to compressive loading, creep, unloading and recovery at multiple load levels corresponding to apparent strains of 2000–25,000 *με*. We found that: the time-dependent response of trabecular bone comprises of both recoverable and irrecoverable strains; the strain response is nonlinearly related to applied load levels; and the response is linked to bone volume fraction. Although majority of strain is recovered after the load-creep-unload-recovery cycle some residual strain always exists. The analysis of results indicates that trabecular bone becomes stiffer initially and then experiences stiffness degradation with the increasing load levels. Steady state creep rate was found to be dependent on applied stress level and bone volume fraction with a power law relationship.

## Introduction

Trabecular bone, a composite cellular material with hierarchical structure, is generally treated as time-independent in biomechanical models.^24^ But in reality its response to mechanical loads is known to be time-dependent.^5^,^13^,^19^,^23^,^29^ Study of this time-dependent behaviour is important in several contexts such as: to understand energy dissipation ability of bone; to understand the age related non-traumatic fractures,^26^ to predict implant loosening due to cyclic load,^30^ to understand progressive vertebral deformity,^25^ and pre-clinical evaluation of total joint replacements.^30^ Consequently, trabecular bone’s time-dependent behaviour has great clinical relevance, but it has received relatively little attention.

A few studies have attempted to relate the creep behaviour with micro-architecture of bone. Kim *et al.* conducted one cycle of load-creep-unload-recovery experiments in which they applied a load corresponding to 2000*μɛ* and found that the samples with thinner trabeculae and greater connectivity were associated with increased logarithmic creep rate.^13^ Novitskaya *et al.* reported the changes in micro-architectural indices evaluated from micro computed tomography (*μ*CT) before and after the creep; the study found that creep induced changes in trabecular separation and structural model index.^23^ Novitskaya *et al.* also found that the steady state creep rate was higher and the final creep strain was larger for samples with low bone volume fraction (BV/TV) (or apparent density).^23^

BV/TV or apparent density have been extensively employed to evaluate the time-independent stiffness of bone,^11^,^14^ which is then used in subject-specific models.^33^ Similar relationships between BV/TV and time-dependent response will permit their application in computational simulations where modelling time-dependent behaviour is important e.g., implant loosening. These relationships need to be considered at multiple loads to incorporate any load-level dependence. Manda *et al.* conducted creep experiments at a single load level (corresponding to a small apparent strain of 2000 *με*) and reported the relationships between BV/TV and linear viscoelasticity for trabecular bone.^19^

Previous studies have shown that under static conditions (or very slow strain rates) the strain in trabecular bone increases non-linearly with applied loads.^10^,^16^,^17^,^21^ However, time dependent behaviour with changing load levels has received limited attention. A few previous studies have considered multiple load levels but different loads were applied to different specimens i.e., each specimen was subjected to a single load level.^4^,^5^,^20^ Bowman *et al*. found a strong power law relationship between the steady state creep rate and the applied stress level, but when they included apparent density into the relation, the fit did not improve, in fact the *r* ^2^ value decreased.^5^ Also, Moore *et al.* related steady state creep rate to applied stress level, but this study also conducted cyclic loading tests on each sample at a single stress level.^20^ Multiple load levels were considered by one recent study in which a mathematical model for the recoverable (or elastic) strain^18^ with respect to load levels was developed; however, while this study alluded to BV/TV relationship with nonlinear viscoelasticity it did not develop it.

In summary, previous studies have shown that under static loading trabecular bone has a non-linear stress–strain behaviour and its time-independent elastic modulus can be related to BV/TV. Therefore, our hypothesis is that the time-dependent behaviour of trabecular bone can also be related to BV/TV and it is not linearly viscoelastic. The aim of this study is to determine how the creep-recovery response varies with load levels and how it can be related to BV/TV.

## Materials and Methods

### Sample Preparation

Bovine proximal femurs, female, under 30 months old, were obtained from a local butcher and stored in a freezer at −20 °C before further preparation. Femoral heads and trochanters were removed using a hacksaw after permitting the bone to thaw at room temperature. Transmission radiographs were taken to identify principal trabecular directions to ensure that samples cored in the following step were aligned along the principal direction. Cylindrical trabecular bone specimens were cored in a hydrated condition, to mitigate against temperature damage, using a 10.7 mm inner diameter diamond-coated coring tool (Starlite, Rosemont, USA). A low-speed saw (Buehler, Germany) was used to trim off growth plate if present and to cut the edges parallel. Thirteen femoral head trabecular bone specimens were obtained from 3 femoral heads and another three from two bovine trochanters (length: 24.8 ± 2.8 mm). The specimens’ dimensions were measured before being glued into brass end-caps using bone cement (Simplex, Stryker, UK) with the assistance of a custom made alignment tool. Effective length for each specimen was calculated as the length between end-caps plus half the length of bone embedded within the endcaps from each side.^12^ Mean effective length was 21.9 ± 2.7 mm.

Each specimen was placed in an epoxy tube filled with phosphate buffered saline (PBS), to ensure that the specimens remain hydrated at all stages of testing. All the specimens were scanned before mechanical testing using micro-computed tomography (*μ*CT) scanner (Skyscan 1172, Bruker, Kontich, Belgium) and the system’s software was used to evaluate bone volume to total volume ratio (BV/TV), which was found to be in the range 15–54%. Degree of Anisotropic (DOA) and Trabecular Thickness (Tb.Th) were also evaluated and found to be in the range 2.04–16.95 and 168.3–277.3 *μ*m, respectively.

### Mechanical Testing

**Figure 1:**
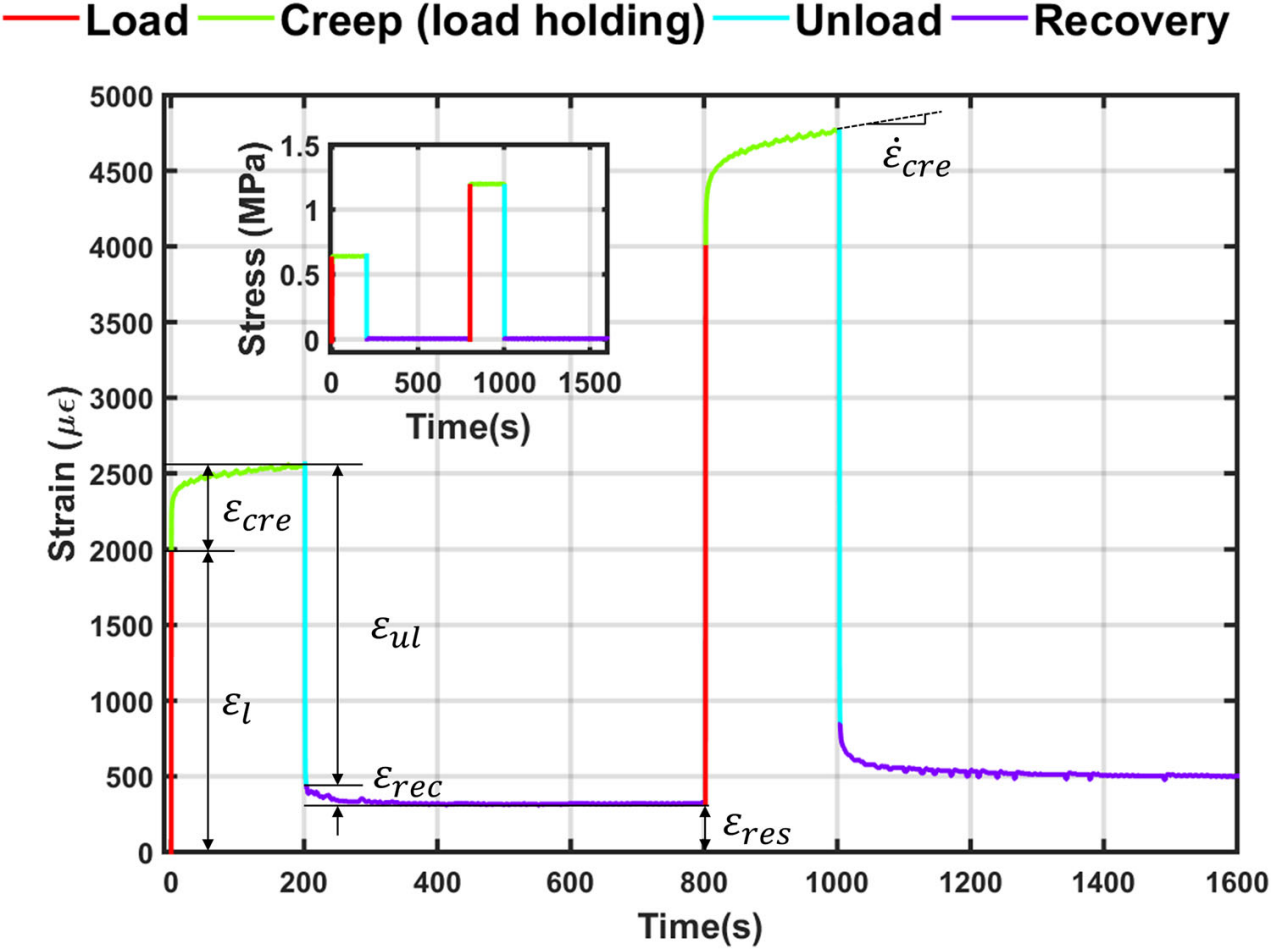
Strain response during MLCUR experiment. Load application is shown in the inset. Only two cycles are shown for clarity.

Mechanical tests were performed at room temperature using Zwick material testing machine (Model Z005/TH2A, Zwick Roell, Herefordshire, UK) with a 5000 N load cell. Each specimen was first preconditioned by subjecting it to 10 cycles of compressive loading with an amplitude of 0.1% apparent strain.^5^ After preconditioning, the specimen was unloaded, removed from the testing machine and allowed to recover for half an hour. Each specimen was then subjected to a compressive multiple load-creep-unload-recovery (MLCUR) cycles. Loading cycles comprised of instantaneous loading strain of 2000 *με*, 4000 *με*, 6000 *με*, 8000 *με*, 10,000 *με*, 15,000 *με*, 20,000 *με*, and 25,000 *με* apparent strains at a rate of 0.01 s^−1^. When the target strain was achieved the corresponding load was maintained for 200 s thereby permitting the specimen to undergo creep. In other words, this was a load-controlled experiment for creep and recovery while instantaneous loading and unloading were displacement controlled. Each loading step was followed by an unloading step to an almost zero force (2 N) and this force was maintained (recovery) for 600 s before the application of the next load cycle. These durations were selected after a number of preliminary tests which showed that the creep rate becomes constant in less than 200 s upon unloading and the recovery curves reach a plateau in less than 600 s. Typical strain response to MLCUR experiment is shown in Fig. 1 (only two cycles are shown for clarity) with the corresponding loading sequence as an inset in the figure. The experiment was stopped immediately if creep strain increased rapidly to beyond 5% in any loading cycle.

In each loading cycle the following strain responses were measured (Fig. 1): *ɛ*_l_ is the instantaneous loading strain, *ɛ*_ul_ is the instantaneous unloading strain, *ɛ*_cre_ is the creep strain accumulated during the plateau loading phase, $\varepsilon_{\text{rec}}$ is the creep strain recovered after load removal, *ɛ*_res_ is the residual strain or the unrecovered strain at the end of each cycle, $\dot{\varepsilon }_{\text{cre}}$ is the steady state creep rate defined as the slope of the linear portion of the secondary creep curve

It is important to note that for a linear viscoelastic material, the ratio *ɛ*_cre_/*ɛ*_l_ will be constant for different load levels and $\dot{\varepsilon }_{\text{cre}}$ will vary linearly with stress level. Also for a viscoelastic material, strain will recover fully if sufficient time is allowed. Strain responses *ɛ*_l_ and *ɛ*_cre_ may include both recoverable and any irrecoverable components, while *ɛ*_ul_ and *ɛ*_rec_ only include the recoverable parts. We evaluated time-varying creep compliance, which is given by

1 $$\varvec{C}_{\text{cre}} \left( t \right) = \varepsilon_{\text{cre}} (t)/\sigma ,$$

where *σ* is the applied stress and *ɛ*_cre_(*t*) is the time-varying strain response due to corresponding constant stress level *σ*.

## Results

In the MLCUR experiments strain output variables as defined in Fig. 1 were measured. Without exception, each specimen exhibited classical rapid primary and slow secondary regimes of creep behaviour across all stress levels.

All 16 specimens could be subjected to a stress level corresponding to 10,000 *με* (cycle 5) without tertiary creep. Four specimens demonstrated tertiary creep^5^ when subjected to stress level corresponding to 15,000 *με* (cycle 6), and only 3 specimens could be subjected to 20,000 *με* (cycle 7) level without tertiary creep. For the sake of completeness only the first 5 cycles were considered for most of the analyses.

**Figure 2:**
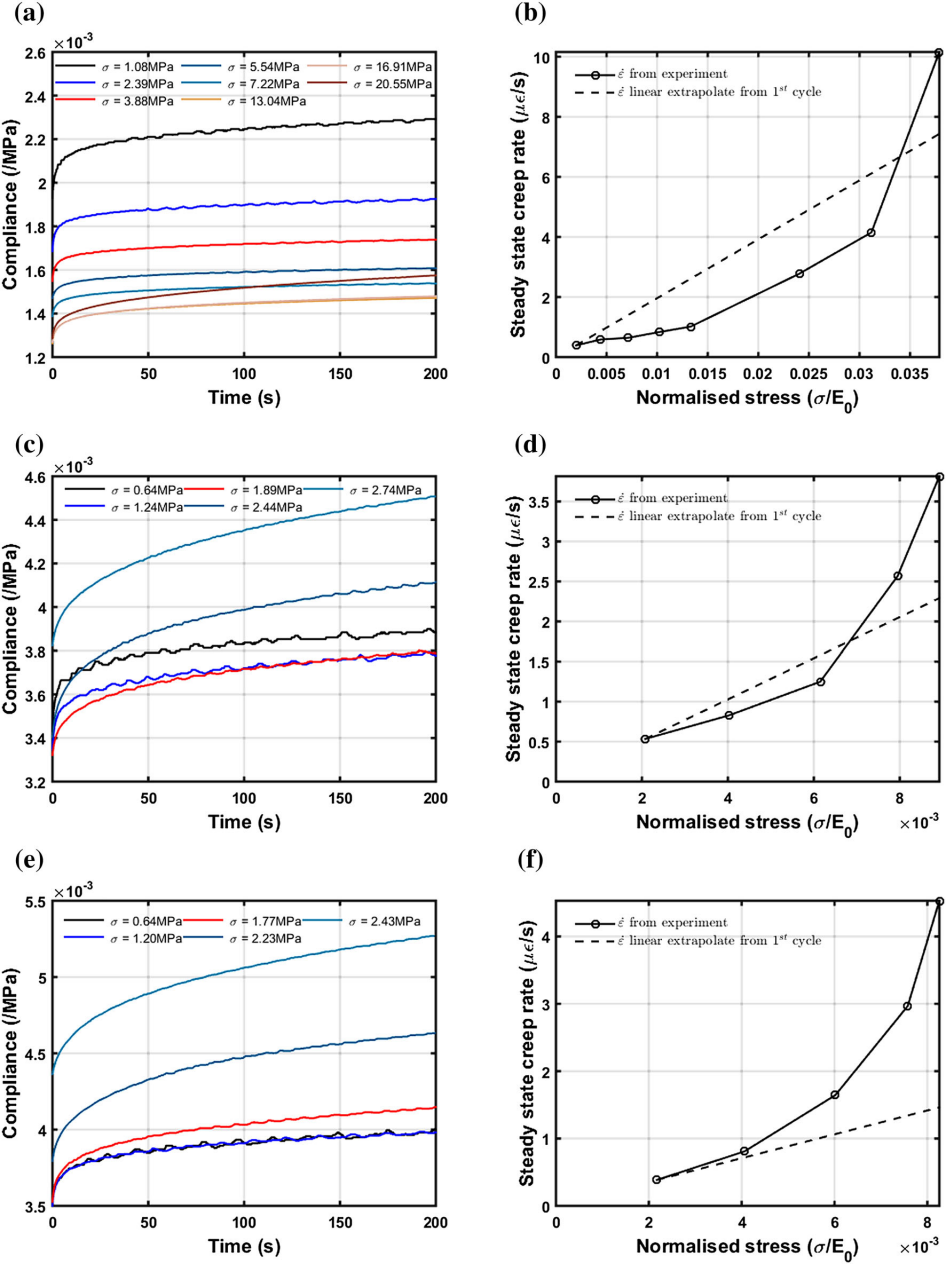
Creep compliance (a, c, e) and steady state creep rate (b, d, f) plots of three typical samples. (a, b) BV/TV = 42.8%, (c, d) BV/TV = 25.1%, (e, f) BV/TV = 18.6%. Dashed line shows extrapolation from the response at the lowest load cycle which is assumed to be linear viscoelastic.

We first examined three typical samples, with a range of bone volume fractions (BV/TV = 42.8, 25.1 and 18.6%) before considering all 16 specimens. Figure 2 shows time-varying creep compliance, $\varvec{C}_{\text{cre}} \left( t \right)$ (Figs. 2a, 2c, and 2e) and the corresponding steady state creep rate (Figs. 2b, 2d, and 2f) at different stress levels for three typical samples with significantly different BV/TV. It can be seen that for the dense sample (Fig. 2a) the time-dependent compliance initially becomes smaller with increasing load levels (the curves at lower stress levels are above those at higher stress levels) and then increases with the load level, at the largest applied stress (20.55 MPa). For the medium BV/TV sample (Fig. 2c), compliance decreases as the stress level is increased from 0.64 to 1.89 MPa but then increases when stress levels are increased to 2.44 MPa and then to 2.74 MPa. This decrease followed by an increase in compliance indicates elastic stiffening followed by elastic softening. For the dense sample, softening occurs at a stress level corresponding to a much higher strain in comparison to the medium BV/TV sample. The trend is followed by the low BV/TV sample (Fig. 2e), which demonstrates softening with increasing load levels right from the beginning.

This stiffening-softening phenomenon can also be seen from the steady state creep rate variation with stress level (Figs. 2b, 2d, and 2f), where we compare the experimentally measured steady state creep rate with the linear extrapolation from the first cycle. If the trabecular bone’s creep behaviour is linear viscoelastic, then the steady state creep rate will be proportional to the normalised stress level. Therefore, we extrapolated the steady state creep rate using the response from the first loading cycle (assumed linear), to predict the linear viscoelastic behaviour of trabecular bone. For a high BV/TV specimen (Fig. 2b), $\dot{\varepsilon }_{\text{cre}}$ is lower than the linear viscoelastic prediction for the first few cycles but higher than the linear viscoelastic prediction at the highest load level applied. For a low BV/TV specimen (Fig. 2f), $\dot{\varepsilon }_{\text{cre}}$ is higher than linear viscoelastic prediction even at lower stress levels while for the medium BV/TV specimen (Fig. 2d) $\dot{\varepsilon }_{\text{cre}}$ is lower than the linear viscoelastic prediction for cycles 2 and 3 and higher than the linear viscoelastic prediction for cycles 4 and 5.

Considering all 16 specimens tested, the steady state creep rate ($\dot{\varepsilon }_{\text{cre}}$) was found to vary from 0.07 to 4.51 *με*/s. The mean $\dot{\varepsilon }_{\text{cre}}$ for load levels corresponding to 2000 *με* and 10,000 *με* were 0.30 *με*/s(±0.12) and 1.84 *με*/s(±1.42), respectively. Regression analysis of the experimental results showed that $\dot{\varepsilon }_{\text{cre}}$ had strong nonlinear (power law) relation with normalised stress level (stress in each cycle divided by the modulus obtained from the first cycle) as defined by Bowman *et al.* 4 The steady state creep rate, $\dot{\varepsilon }_{\text{cre}}$, was also found to have a strong relationship with BV/TV. The best fit equation was found to be

2 $$\dot{\varepsilon }_{\text{cre}} = 0.003103 \sigma^{1.256} ({{\text{BV}} \mathord{\left/ {\vphantom {{\text{BV}} {\text{TV}}}} \right. \kern-0pt} {\text{TV}}})^{ - 3.469} ,$$

where $$\dot{\varepsilon }_{\text{cre}}$$ is in *με*/s, σ is in MPa and BV/TV is the bone volume fraction ($$r^{2} = 0.74, p < 0.001)$$.

**Figure 3:**
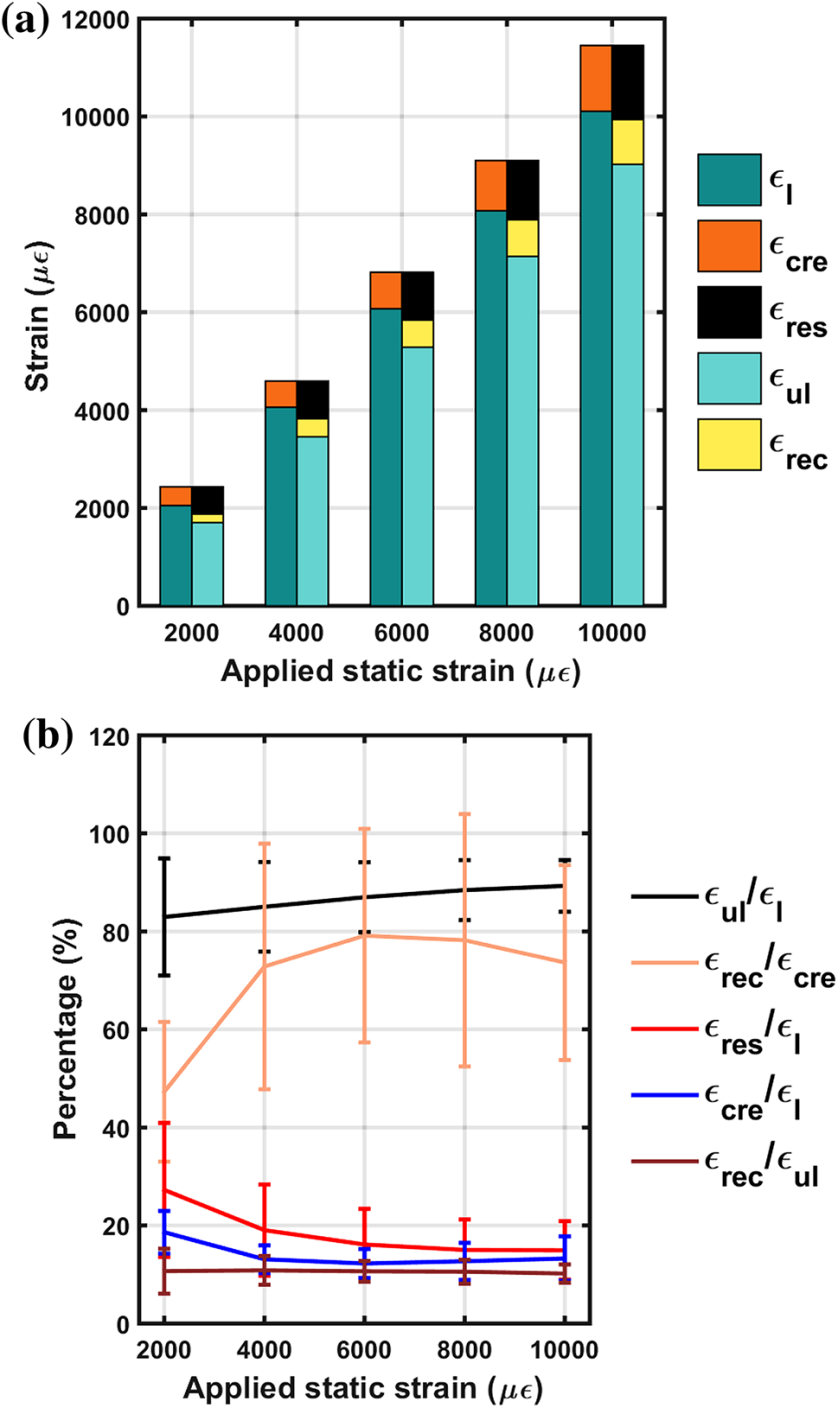
Measured strain response during MLCUR experiments. (a) Mean strain responses for the 16 samples tested for the five creep-recovery cycles; (b) Strain ratios and their variation for different creep-recovery cycles. (Large BV/TV variation results in large variation for some of the ratios).

Figure 3a shows a bar plot of the measured mean strain responses for different load cycles for all samples tested: initial loading strain (*ɛ*_l_), increase in creep strain during stress holding cycle (*ɛ*_cre_), instantaneous unloading strain (*ɛ*_ul_), decrease in strain during recovery (*ɛ*_rec_) and residual strain (*ɛ*_res_). Figure 3b shows the ratios of different strain responses against load cycles.

The ratio of *ɛ*_ul_/*ɛ*_l_ increased with increasing corresponding strain level for all 16 specimens (Fig. 3b). Majority of instantaneous strain was recovered immediately upon unloading (average 86.5% for pooled data, *r* ^2^ = 0.99).

Our results showed that some residual strain, *ɛ*_res_, always exists at the end of 600 s of recovery after every unloading cycle and for all 16 specimens. The mean residual strain (Fig. 3a) (±SD) at the lowest load level corresponding to instantaneous loading strain of 2000 *με* was 542 *με* (±255) and for higher load level corresponding to instantaneous loading strain of 10,000 *με* was 1523 *με* (±604). The ratio of *ɛ*_res_/*ɛ*_l_ was found to decrease with increasing load level (Fig. 3b) indicating that residual strain does not increase proportionally with load level.

Creep strain during stress holding cycle, *ɛ*_cre_, was found to increase with increasing load level (Fig. 3a). From cycle 1 to cycle 5 it increased from 377*µε* (±84) to 1365*µε* (±498), however, the ratio *ɛ*_cre_/*ɛ*_l_ was found to decrease after the first load cycle after which it remained almost constant (Fig. 3b).

As expected, $\varepsilon_{\text{ul}}$ and *ɛ*_rec_ both increased with increasing load level (Fig. 3a), from 1718 *με* (±266) and 180 *με* (±45) to 9048 *με* (±544) and 928*µε* (±157), respectively, and had relatively constant ratio of *ɛ*_rec_/*ɛ*_ul_ (Fig. 3b) indicating that, as would be expected, the unloading phase is viscoelastic.

As mentioned above both *ɛ*_cre_ and *ɛ*_rec_ increased with increasing load levels (Fig. 3a), however, the ratio, *ɛ*_rec_/*ɛ*_cre_, had a very interesting trend—it first increased with load level and then decreased at higher load level (Fig. 3b). This trend again indicates elastic stiffening is followed by elastic softening with increasing load levels as demonstrated earlier by individual samples.

## Discussion

Our study shows that residual strain arises even at low load levels, trabecular bone response is not linear viscoelastic and that bone demonstrates stiffening followed by elastic softening with increasing load levels.

It is now generally accepted that the yield strain of trabecular bone is independent of BV/TV.^1^,^15^ Typically, trabecular bone macroscopically yields below 0.8% strain in compression.^21^ Therefore, we applied compression forces equivalent to different strain levels (from 2000 *με* to 25,000 *με*), to examine the time-dependent behaviour of trabecular bone in pre- and post-yield regimes.

Examination of creep compliance curves for different samples showed that they vary with load levels. The samples with medium BV/TV showed an initially decreasing and then increasing creep compliance with increasing stress. This indicates that the samples first becomes stiffer and then experience softening (stiffness degradation). High BV/TV samples demonstrated decreasing creep compliance with stress indicating stiffening and an increase is observed only at much higher stress levels. For low BV/TV samples compliance increased with stress levels indicating softening from the start. This behaviour was also demonstrated by steady state creep rate comparison with creep rate linearly extrapolated from the first cycle: for low BV/TV samples the steady state creep rate was higher than linear extrapolation throughout; for high BV/TV samples it was below the linearly extrapolated values for most stress levels; and for medium BV/TV samples it was initially below the linear extrapolation and then above at higher stress levels. On average the ratio *ɛ*_rec_/*ɛ*_cre_ initially increases and then decreases with stress level again demonstrating stiffening and softening behaviour, which has also been reported through nonlinear time-dependent constitutive models.^18^ A time-dependent constitutive model was previously developed by Fondrk *et al*. for cortical bone which incorporated stiffness degradation via damage but did not have stiffening demonstrated in this study.^8^ It is, however, not apparent why this occurs; it could be due to the reorganisation of ultrastructural components (i.e., mineral and collagen) in the bone matrix that make it stiffer initially followed by damage and buckling of trabeculae causing softening. Although the movement of collagen is constrained by mineral,^2^ sliding of collagen fibrils plays an important role in the time-dependent properties of bone.^4^,^27^,^32^ It is likely that collagen fibrils initially reorganise when the load levels are increased to resist deformation and maintain network integrity, and at larger loads micro-damage breaks this integrity.

Kim *et al.* reported mean $\dot{\varepsilon }_{\text{cre}}$ of 0.22 *με*/s when the specimens were compressed at stress levels corresponding to 2000 *με*.^13^ This compares well with the mean value (0.30 *με*/s) found in this study. We also found that increased stress level does not result in a linear increase of $\dot{\varepsilon }_{\text{cre}}$ which would be expected from a linear viscoelastic material, e.g., at stress level corresponding 10,000 *με* the steady state creep rate (1.84 *με*/s) was more than five times of the value at 2000 *με*. The steady state creep rate can be reasonably well related to stress level and BV/TV.

Ratio of *ɛ*_ul_/*ɛ*_l_, was found to be high (>80%) and increase slightly with increasing load levels. For a viscoelastic material in a creep-recovery experiment (instantaneous loading and unloading) this ratio is unity. The ratio *ɛ*_ul_/*ɛ*_l_ < 1 indicates presence of irrecoverable strains arising during the loading phase. Yamamoto *et al.* found little difference between instantaneous loading and unloading strains.^31^ Kim *et al.* considered a single load level and found that 92.3% of strain was recovered immediately upon unloading.^13^ Kim *et al.* suggested that the difference between *ɛ*_ul_ and *ɛ*_l_ implies a reorganisation of micro- or ultra-structural components of the bone matrix caused by compressive creep and this reorganised state is not fully released upon unloading.^13^ Smallest *ɛ*_ul_/*ɛ*_l_ ratio at low load level indicates that most reorganisation of the bone matrix happens at its first loading experience. The fact that the majority of the strain was recovered in the unloading phase was found to be true for all specimens and for all load cases.

Strain, *ɛ*_res_ always exists even at low load levels, which implies that certain amount of irrecoverable strain is generated during loading and load holding. This study found that the average ratio of residual strain to loading strain (*ɛ*_res_/*ɛ*_l_) varied from 26% in the first loading cycle to 15% in the fifth loading cycle. Yamamoto *et al*. measured *ɛ*_res_ of human L3 vertebral trabecular bone and reported mean values of 515 *με* and 1565 *με* for load levels corresponding to 750 *με* and 1500 *με*, respectively i.e., *ɛ*_res_/*ɛ*_l_ ratios of 69 and 104%.^31^ Similarly, Kim *et al.* reported an average *ɛ*_res_/*ɛ*_l_ value of 90% at load level corresponding to 2000 *με*.^13^ In both these studies the load holding time was much longer—Yamamoto *et al.* held the load for around 35 h while Kim *et al.* held it for 2 h. Yamamoto *et al.* extrapolated that the residual strain may fully recover in sufficiently long time (20 times the load holding time).^31^ Our tests showed that the decrease in *ɛ*_res_ beyond 600 s was negligible i.e., these residual strains were largely irrecoverable. Large *ɛ*_res_/*ɛ*_l_ ratios in the above cited studies in comparison to ours indicate that irrecoverable strains accumulate during load holding.

The ratio *ɛ*_rec_/*ɛ*_ul_ was found to be constant in our study indicating that the unloading phase is viscoelastic. The ratio *ɛ*_cre_/*ɛ*_l_ was found to decrease with increasing applied stress level initially and then become almost constant (it slightly increased at higher stress levels in samples which were tested beyond the 5 cycles). We found *ɛ*_cre_/*ɛ*_l_ > *ɛ*_rec_/*ɛ*_ul_ for all stress levels indicating presence of irrecoverable strains arising in the loading and load holding phases.

Our work suffers from a number of limitations. Firstly, all the tests were conducted at room temperature; creep behaviour has been reported to be temperature dependent.^3^,^4^ Secondly, the identification of instantaneous (loading and unloading) strain responses from the time-dependent strain response in MLCUR experimental curves was done using the loading platens of the machine rather than an extensometer attached to the central region with a more homogeneous mechanical environment, which may result-in small errors in the analysis of the results. Thirdly, a small force of 2 N was used during recovery phase to make sure that the end-caps were in contact with the load applicator to facilitate the measurement of the strain response. We believe the effect of this small load is negligible on the measured response.

An important clinical implication of the present study relates to the possible role of creep mechanisms and deformations in non-traumatic bone fractures. Non traumatic vertebral fracture present as shortening or height loss of bone without obvious trauma, and the progression is very slow and occurs gradually over a long period.^22^,^25^ It has been suggested that during normal daily activities, strain in bone usually does not exceed 3000 *με*.^6^ However, strain concentrations can arise at the bone implant interface e.g., when fractures are treated using external fixators.^7^ Also results from our study show that residual strain exists even at low stress level (equivalent to 2000 *με*), and it is accumulated with increasing stress levels. Trabecular bone with relative low BV/TV has larger value of steady state creep rate. Our study also shows that low BV/TV bone demonstrates stiffness degradation (or starts softening) even at low stress levels corresponding to 2000–4000 *με*. The BV/TV range considered by this study was 15–54%; previous studies have shown that BV/TV for human lumber spine can be around 8%,^9^ resulting in stiffness degradation at even lower loads. It has been previously suggested that creep deformity could accumulate over time in elderly human bones due to their reduced ability to remodel.^28^ Findings from current study indicate that elderly people who suffer from osteoporosis and consequently have low BV/TV are at greater risk of non-traumatic fractures even under normal physiological loads.
